# Documentation of a novel *FBP1* gene mutation in the Arabian ethnicity: a case report

**DOI:** 10.1186/s13256-024-04448-9

**Published:** 2024-04-09

**Authors:** Maher Almousa, Mohammad Aljomaa, Shekhey Hamey, Diana Alasmar

**Affiliations:** 1 Faculty of Medicine, Hama University, Hama, Syria; 2 Department of Gastroenterology, Aleppo University Hospital, University of Aleppo, Aleppo, Syria; 3 Faculty of Medicine, Damascus University, Damascus, Syria; 4 Department of Pediatrics, University Children Hospital, Damascus University, Damascus, Syria

**Keywords:** Fructose-1,6-bisphosphatase deficiency, Gluconeogenesis, *FBP1* gene, Novel mutation, Arab population, Case report

## Abstract

**Background:**

Fructose-1,6-bisphosphatase deficiency is a rare autosomal recessive disorder characterized by impaired gluconeogenesis. Fructose-1,6-bisphosphatase 1 (*FBP1*) mutations demonstrate ethnic patterns. For instance, Turkish populations commonly harbor exon 2 deletions. We present a case report of whole exon 2 deletion in a Syrian Arabian child as the first recording of this mutation among Arabian ethnicity and the first report of *FBP1* gene mutation in Syria.

**Case presentation:**

We present the case of a 2.5-year-old Syrian Arab child with recurrent hypoglycemic episodes, accompanied by nausea and lethargy. The patient’s history, physical examination, and laboratory findings raised suspicion of fructose-1,6-bisphosphatase deficiency. Whole exome sequencing was performed, revealing a homozygous deletion of exon 2 in the *FBP1* gene, confirming the diagnosis.

**Conclusion:**

This case highlights a potential novel mutation in the Arab population; this mutation is well described in the Turkish population, which suggests potential shared mutations due to ancestral relationships between the two ethnicities. Further studies are needed to confirm this finding.

## Background

Fructose-1,6-bisphosphatase deficiency is a rare autosomal recessive inherited disorder that results in impaired gluconeogenesis. This disorder is caused by mutations in the gene that encodes for fructose-1,6-bisphosphatase 1 (*FBP1*). It was first described in 1970 as recurrent episodes of fasting hypoglycemia and metabolic and lactic acidosis manifesting as hyperventilation, apneic spells, seizures, or coma, which take place usually early in childhood [1, 2]. Episodes might be triggered by fever, fasting, decreased oral intake, vomiting, infections, and ingestion of large amounts of fructose, untreated patients are at risk of multiorgan failure (especially liver and brain), and morbidity and mortality are high. Reye syndrome-like presentations have been reported [2]. With early diagnosis and exact management, the long-term prognosis is excellent, which highlights the importance of early diagnosis [3]. Fructose-1,6-bisphosphatase gene is composed of eight exons located on chromosome 9q22.2-q22.3 [4]. Many *FBP1* gene mutations have been described for each ethnic group; exon 2 deletion is a common mutation among Turkish populations, and has been reported in Armenian populations as well [5, 6]. Here we report an *FBP1* gene mutation in a Syrian Arabian child where DNA sequencing of the *FBP1* gene identified a homozygous 170 base pair deletion encompassing whole exon 2 (previously termed exon 1) of the *FBP1* gene (Chr9: 94,639,141–94,639,310). This mutation has not been reported before among people of Arabian ethnicity, and this is the first documentation of the *FBP1* mutation in Syria.

### Case presentation

**Fig. 1:**
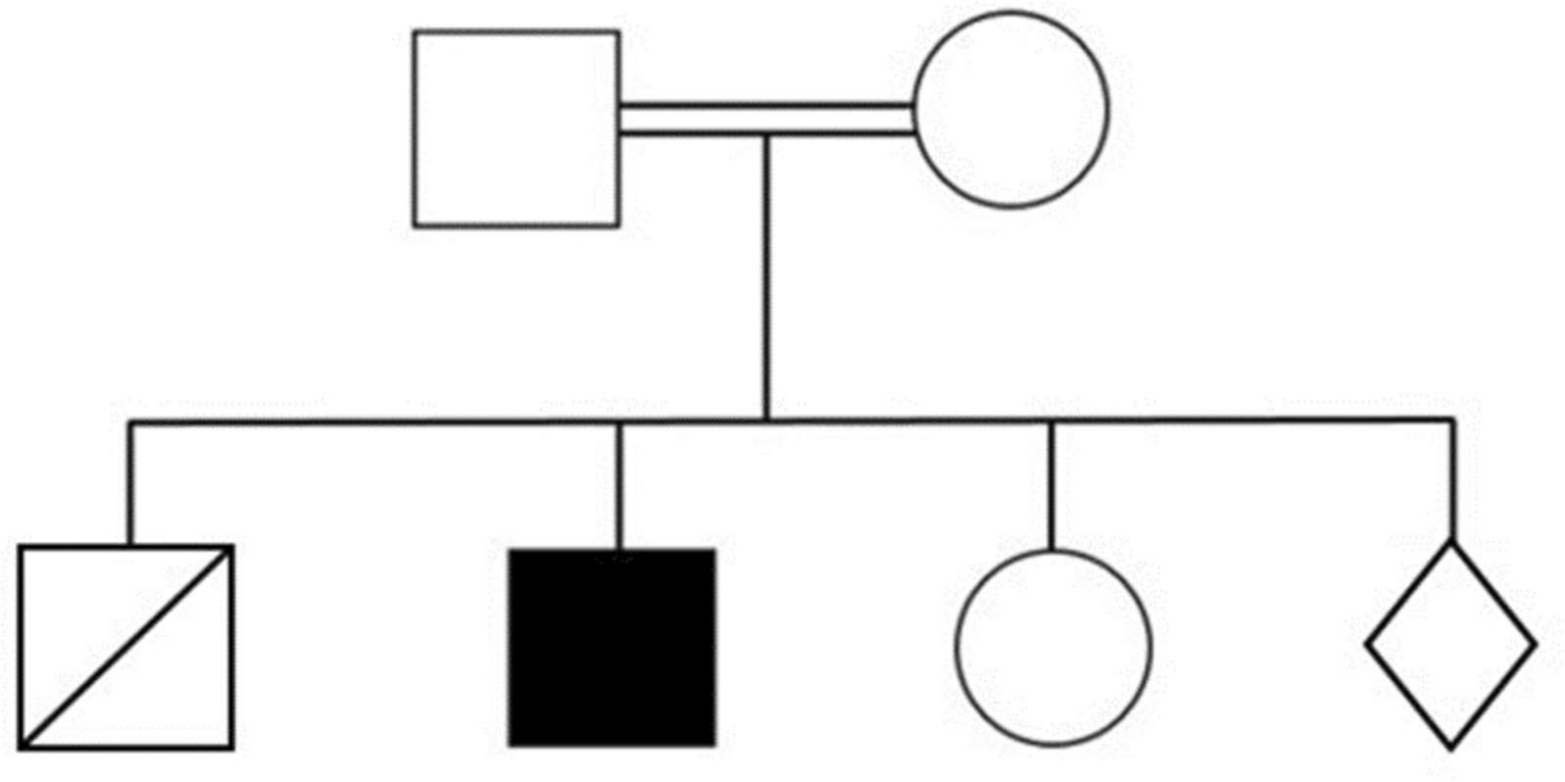
Pedigrees of the family

A 2.5-year-old Syrian Arab child presented to the outpatient clinic in our hospital with nausea, lethargy, and an acutely sick appearance. The patient has a 6-month history of recurrent episodes of hypoglycemia sometimes accompanied by fever and intestinal infection, and required hospitalization. The patient was born by cesarean delivery after normal history of pregnancy; he weighed 2400 g at birth. The patient was previously diagnosed with growth hormone deficiency on the basis of a laboratory evaluation (Table 1) and was administered replacement therapy 6 months before presentation. Before coming to our hospital, the patient was admitted to a local hospital due to episodes of vomiting, seizures, and deteriorating condition, where he was treated with intravenous fluids by glucose 5% solution, antibiotics, and antiemetics. Family history showed a kinship relationship between the parents; they have another girl and lost a boy 15 days after birth due to dehydration and poor breastfeeding, and the mother is currently pregnant (Fig. 1). Otherwise, his family and psychosocial history were insignificant as the parents were healthy and no abnormal conditions in his family were reported. Physical examination revealed asthenia and hepatomegaly. Laboratory evaluation results are presented in Tables 2, 3 and 4. Cardiac ultrasound and electrocardiogram were normal, whereas abdomen ultrasound showed homogeneous hyperechoic hepatomegaly 15.7 cm in the right anterior axillary line. Fructose-1,6-bisphosphatase deficiency was suspected. As testing of *FBP1* activity in liver or mononuclear white blood cells was not available, genetic testing was suggested. The patient underwent whole exome sequencing, showing a homozygous 170 base pair deletion encompassing whole exon 2 of the *FBP1* gene (Chr9: 94,639,141–94,639,310). On the basis of the genetic test, the 6-bisphosphatase deficiency was confirmed. Follow-up included recommendations to avoid fasting particularly during febrile episodes, frequent feeding, the use of slowly absorbed carbohydrates (such as uncooked starch), and gastric drip, if necessary. Three months of follow-up revealed that the patient’s condition improved significantly with the disappearance of episodes of hypoglycemia. Table 5 presents laboratory evaluation for follow-up of the patient.

**Table 1:** Laboratory test results before presenting

Test	Glucose	Insulin	Cortisol	Ketone bodies	Growth hormone
Result	36	0.2	21.8	Negative	2.86
Normal value	(65–110) mg/dl	(0–30) uIU/ml	(5–25) Ug/dl	(0.03–0.3) mmol/l	(0–7) ng/ml

**Table 2:** Laboratory test results at presenting

Test	GRA	WBC	CRP	UA	AST	ALT	CREA	BUN	GLU
Result	55	7900	0.09	5	48	61	049	11	60–240
Unit	%	10^3^/ml	m/l	mg/dl	U/l	U/l	mg/dl	mg/dl	mg/dl

*GRA* glucocorticoid-remediable aldosteronism, *WBC* white blood cells, *CRP* C-reactive protein, *UA* uric acid, *AST* aspartate aminotransferase, *ALT* alanine transaminase, *CREA* creatinine, *BUN* blood urea nitrogen, *GLU* glucose

**Table 3:** Laboratory test results at presenting

Cl	K	Na	KBU	KBB	Lactate	GLU*
98.5	3.2	138	Negative	Positive	36	106
mmol/l	mmol/l	mmol/l			mg/dl	mg/dl

*KBU* ketone bodies in urine, *KBB* ketone bodies in blood, *GLU* glucose

*After 18 hours fasting

**Table 4:** Laboratory test results at presenting

PTT	PT	ALP	CHOL	TG	P	Ca	CK	Alb	TP
30	100	137	133	301	3.1	9.61	43	3.9	5.38
27 seconds	%	U/l	mg/dl	mg/l	mg/dl	mg/dl	U/l	g/dl	g/dl

*PPT* partial thromboplastin time, *PT* prothrombin time, *ALP* alkaline phosphatase, *CHOL* cholesterol, *TG* triglyceride, *P* phosphate, *Ca* calcium, *CK* creatine kinase, *Alb* albumin, *TP* total protein

**Table 5:** Laboratory test results tow months after treatment

Test	Result	Units
Glucose	105	mg/dl
Triglyceride	75	mg/l
Cholesterol	178	mg/dl
Uric acid	3.5	mg/dl
Lactate	28.8	mg/dl
KBB	0.11	mg/dl
NH_4_	42	mg/dl
pH	7.4	
pCO_2_	33.7	mmHg
HCO_3_	22.4	mmol/l
BE	−1	mmol/l

*NH*_*4*_ Ammonia, *HCO*_*3*_ Bicarbonate, *pH* potential of Hydrogen

## Discussion

This case presents the first reported *FBP1* gene mutation in Syria, and report a novel *FBP1 *gene mutation in the Arabian population. The *FBP1* gene is located on chromosome 9 and encodes the fructose-1,6-bisphosphatase enzyme, which plays a crucial role in the regulation of glucose metabolism [1, 2].

In this case report, whole exome sequencing revealed a homozygous 170 base pair deletion of exon 2 of the *FBP1* gene (Chr9: 94,639,141–94,639,310) in a 2.5-year-old Syrian Arab child. This deletion results in a frame shift mutation, leading to a premature stop codon and the production of a truncated nonfunctional fructose-1,6-bisphosphatase enzyme [3].

The molecular basis of *FBP1* deficiency lies in the disruption of the gluconeogenic pathway. Fructose-1,6-bisphosphatase is a key enzyme in this pathway, responsible for the conversion of fructose-1,6-bisphosphate to fructose-6-phosphate, which is an essential step in gluconeogenesis. Mutations in the *FBP1* gene impair the enzymatic activity of fructose-1,6-bisphosphatase, leading to decreased glucose production and subsequent hypoglycemia [2].

*FBP1* mutations among the Arabian population were identified in a study conducted on Arab patients with fructose-1,6-bisphosphatase deficiency. Two novel mutations were identified in the *FBP1* gene. A novel six-nucleotide repetitive insertion, c114_119dupCTGCAC, was identified in patients from three families. This mutation encodes for the duplication of two amino acids (p.Cys39_Thr40dup) in the N-terminal domain of *FBP1*. Another novel nonsense c.841G > T mutation encoding for a p.Glu281X truncation in the active site of *FBP1* was discovered in patients from two families. These newly identified mutations were predicted to produce FBP1 deficiency and were the only known genetic causes of *FBP1* deficiency in Arab patients [6].

Importantly, the identification of this specific mutation in the Syrian Arab population highlights the ethnic diversity of *FBP1* mutations. Previously, this mutation has been reported in the Turkish population. The presence of the same mutation in different ethnic groups suggests either a common ancestral origin or a recurrent mutational event. The occurrence of *FBP1* deficiency-associated mutations in distinct populations underscores the significance of genetic heterogeneity and highlights the need for expanded genetic screening programs across diverse ethnic backgrounds [3, 6].

Understanding the molecular basis of *FBP1* mutations is crucial for accurate diagnosis, prognosis, and management of affected individuals. Molecular genetic testing, such as whole exome sequencing, enables the identification of specific mutations, facilitating precise diagnosis and appropriate management strategies tailored to the individual patient’s needs [4].

The identification of a homozygous exon 2 deletion in the *FBP1* gene in a Syrian Arab child with *FBP1* deficiency highlights the ethnic diversity of *FBP1* mutations. This case emphasizes the importance of considering population-specific variations in the diagnosis and management of rare genetic disorders. Further studies and collaborative efforts involving diverse populations are necessary to unravel the complete spectrum of *FBP1* mutations, enabling comprehensive genetic counseling, early detection, and optimal management of affected individuals worldwide.

## Conclusion

*FBP1* exon 2 deletion mutation is well described in the Turkish population but not in the Arabian population. This suggests potentially shared mutations due to ancestral relationships between the two ethnicities. We suggest conducting further studies to confirm this finding.

## Data Availability

Not applicable.

## Abbreviation

*FBP1*: Fructose-1,6-bisphosphatase 1
